# MRD in AML: The Role of New Techniques

**DOI:** 10.3389/fonc.2019.00655

**Published:** 2019-07-23

**Authors:** Maria Teresa Voso, Tiziana Ottone, Serena Lavorgna, Adriano Venditti, Luca Maurillo, Francesco Lo-Coco, Francesco Buccisano

**Affiliations:** ^1^Department of Biomedicine and Prevention, Tor Vergata University, Rome, Italy; ^2^Santa Lucia Foundation, IRCCS, Neuro-Oncohematology, Rome, Italy

**Keywords:** NGS, digital droplet PCR, multiparametric flow-cytometry, MRD, AML

## Abstract

In the context of precision medicine, assessment of minimal residual disease (MRD) has been used in acute myeloid leukemia (AML) to direct individual treatment programs, including allogeneic stem cell transplantation in patients at high-risk of relapse. One of the limits of this approach has been in the past the paucity of AML markers suitable for MRD assessment. Recently, the number of biomarkers has increased, due to the identification of highly specific leukemia-associated immunophenotypes by multicolor flow-cytometry, and of rare mutated gene sequences by digital droplet PCR, or next-generation sequencing (NGS). In addition, NGS allowed unraveling of clonal heterogeneity, present in AML at initial diagnosis or developing during treatment, which influences reliability of specific biomarkers, that may be unstable during the disease course. The technological advances have increased the application of MRD-based strategies to a significantly higher number of AML patients, and the information deriving from MRD assessment has been used to design individual post-remission protocols and pre-emptive treatments in patients with sub-clinical relapse. This led to the definition of MRD-negative complete remission as outcome definition in the recently published European Leukemianet MRD guidelines. In this review, we summarized the principles of modern technologies and their clinical applications for MRD detection in AML patients, according to the specific leukemic markers.

## Introduction

Acute myeloid leukemia (AML) is a heterogeneous group of diseases with variable response to therapy, due to intrinsic genetic complexity, present at diagnosis, and/or developing during disease evolution (1, 2). For these reasons, characterization of molecular and phenotypic AML profiles is essential at the time of initial presentation, with the objective to design patient- and disease-specific strategies aimed to prevent disease relapse and improve long-term outcome.

**Figure 1:**
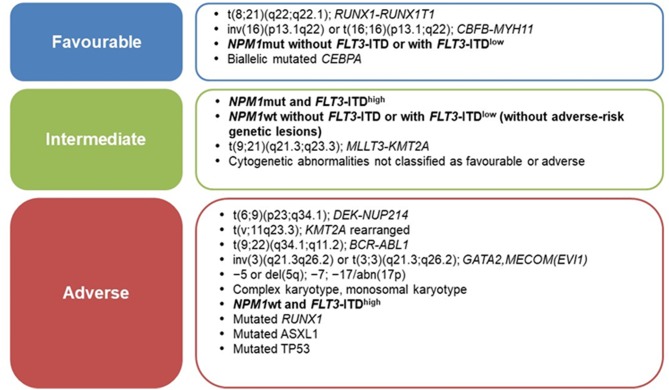
ELN-2017 risk stratification of AML by genetic abnormalities [Adapted from Dohner et al. (6)].

Current prognostic assessment of AML depends on several factors (3, 4). Cytogenetics and mutational profile allow to stratify AML into prognostic subgroups associated with significantly different complete remission (CR), relapse and overall survival (OS) rates (5); thus, genetic characteristics of leukemic cells are increasingly used to guide treatment approaches (3, 6). In 2010, the European LeukemiaNet (ELN) defined the first genetic-based stratification system for AML and recently published a revised version, refining the definition of three prognostic subgroups (favorable, intermediate, adverse), rather than the previous four groups (6, 7) (Figure 1). Among major changes made in the revised system is the quantification of *FLT3*-ITD allelic burden, defined by the ITD allelic ratio (AR), which has been associated to the risk of relapse (8). In this context, *NPM1*-mutated AML with *FLT3*-ITD AR < 0.5 (*FLT3*-ITD*low*) are classified as prognostically favorable, similar to *NPM1*-mutated/*FLT3* wild-type AML (6). Notably, this is the first time that *FLT3*-ITD mutated AML are classified as “favorable” (9). By contrast, *NPM1* wild-type AML with *FLT3*-ITD AR >0.5 (*FLT3*-ITD*high*) are classified as “adverse-risk,” while AML *NPM1*-mutated/*FLT3*-ITD*high* are considered “intermediate-risk” (6) (Figure 1).

Prognostic assessment of AML at diagnosis has strong clinical relevance, allowing for instance to allocate patients with high-risk disease to receive allogeneic stem cell transplantation (allo-SCT) in first remission as part of frontline therapy (10, 11), while patients with favorable-risk AML would not be candidates to allo-SCT (6). Conversely, the indication for allo-SCT in first complete remission for the large subset of patients with intermediate-risk disease is currently a matter of debate (12). These figures may further change in the near future due to the incorporation of targeted treatments added to conventional chemotherapy, as the recently approved FLT3 inhibitors midostaurin or quizartinib (13, 14), which may change risk-stratification according to *FLT3* mutation status.

Recent technological advances have contributed to increase the list of AML mutations associated to adverse prognosis. In particular, in the past decade, the advent of Next Generation Sequencing (NGS) technologies led to the identification of novel molecular abnormalities that shed new light into the pathogenesis and prognosis of the disease, and have been therefore, incorporated in the revised ELN guidelines (6, 15, 16).

Despite improved genetic classification and CR rates close to ~80%, more than 50% of adult patients with AML will undergo disease relapse after initial treatment (12, 17), due to the emergence of resistant clones surviving therapy (18). In fact, although an accurate quantification of blasts may be provided by optical microscopy when declaring morphological CR (mCR), a relevant amount of residual leukemic cells, estimated to range from 10^10^ to 10^12^ may still persist in a normal adult (19). A precise estimate of residual disease below the threshold of mCR represents a critical issue in prognostic assessment and in the post-remission decision-making process in AML. This is the purpose of techniques aiming at minimal residual disease (MRD) detection (20). However, since the capability to detect leukemic cells depends on the methodology used, the term “measurable” residual disease has been recently proposed to indicate the levels of leukemic cells detectable by modern technologies, which are characterized by very high sensitivity (19). The advantage of identifying by molecular methods MRD levels predictive of clinical relapse has been proven for acute promyelocytic leukemia (APL), where a positive *PML-RARA* test after the end of consolidation therapy is invariably associated to overt relapse (21). As a consequence, pre-emptive treatment of molecular relapse is currently recommended as standard practice in APL and is being now considered in some specific non-APL AML subsets (22).

In this context, the updated ELN recommendations introduced the concept of MRD-negative mCR as a new outcome definition (6), in cases where a given genetic or immunophenotypic aberration present at diagnosis is no longer detectable by high sensitivity multiparametric flow-cytometry (MFC), or reverse transcriptase-quantitative PCR (RT-qPCR) (6, 23). Following the definition of this new outcome, the AML ELN MRD working group felt the need for the first consensus document on MRD standardization/harmonization in AML (24). According to the panel, MRD measurement provides an objective methodology to establish a deeper remission status in AML treatment, whatever the method employed. The ELN consensus suggests that efforts should be directed to assign to individual patients a reliable tool to monitor MRD and estimate the risk of relapse. More specifically, AML subgroups including APL, core binding factor (CBF) AML, and *NPM1*-mutated AML should be monitored by RT-qPCR, whereas the use of MFC is strongly recommended in all other AML subgroups (23, 24).

In this review, we will focus on recent methodological and conceptual advances in the field of MRD assessment in AML, and their inclusion in the decision-making process of personalized treatment.

## RT-qPCR

In AML, molecular MRD monitoring includes quantification of *PML-RARA* *(**25*, *26**), RUNX1-RUNX1T1* *(**27**), CBFB-MYH11* *(**28**)*, and mutated-*NPM1* (29, 30). RT-qPCR methods for the above fusion genes have been standardized by the Europe Against Cancer (EAC) consortium, and are widely used by hematology laboratories worldwide (31). The advantages of RT-qPCR over nested-PCR include high specificity and sensitivity for leukemic cells, decreased contamination risk, better evaluation of the quality of RNA, and potential to assess the kinetics of MRD longitudinally. As a consequence, this approach has had wide application in routine patients' care. The clinical importance of MRD monitoring has been best established in APL, where achievement of molecular remission in the bone marrow (BM) after consolidation treatment is regarded as a therapeutic objective (26) and a powerful independent predictor of disease relapse (21). The role of the best time-point for MRD studies is critical in AML, and in particular in APL, both in patients treated with ATRA-chemotherapy and in those treated with arsenic-trioxide/ATRA regimens (32). As of CBF fusion transcripts (*RUNX1-RUNX1T1* and *CBFB-MYH11)*, some studies have demonstrated the prognostic value of MRD detection and quantification after induction therapy (27, 28), albeit these transcripts can persist in CR in the long-term, without effects on treatment outcome (33). Moreover, Jourdan et al. (27), demonstrated that the 3-log MRD CBF fusion transcripts reduction can be used to discriminate high-risk from low-risk patients. Interestingly, Yin et al. (34), showed that BM and peripheral blood (PB) levels of *RUNX1-RUNX1T1* and *CBFB-MYH11* transcripts after consolidation therapy and during remission were predictive of relapse risk. In this context, PB was shown to be more sensitive and informative as starting material for MRD detection, as compared to BM.

Similar to *PML-RARA, NPM1* mutations, present in founder clones in 40–50% of AML patient with normal karyotype (35) are stable during the course of the disease, therefore representing an ideal leukemia-specific target for MRD assessment (29, 36, 37). Following the first application of specific RT-qPCR assays to evaluate the mutation kinetics (38), several studies have investigated the clinical implications of *NPM1* monitoring (29, 39, 40). In particular, Ivey et al. showed in a large prospective study that detection of persistent molecular MRD after the second chemotherapy cycle has clinical relevance (29). In fact, detection of *NPM1*-mutated transcripts at this time-point was associated with a significantly higher relapse risk and poorer survival rates, independent of other known prognostic factors (29).

Based on these findings, the ELN Working Party consensus document on MRD in AML (6), indicates that molecular assessment for *NPM1* mutations, *RUNX1-RUNX1T1, CBFB-MYH11*, and *PML-RARA* fusion transcripts, should be performed at diagnosis, at least after two cycles of induction/consolidation therapy, and every 3 months, for 24 months after the end of treatment. Monitoring of *NPM1* mutated transcripts may be more informative when performed in PB as compared to BM (29). As to cost-effectiveness, MRD monitoring after 2 years of follow-up should be decided based on the relapse risk of the specific AML subtype (24).

Conversely, the reliability of *WT1* expression analysis as MRD marker has remained more controversial, due to the low sensitivity and specificity of the molecular assays, based on arbitrary cut-off levels for *WT1* overexpression in malignant cells, as compared to normal hematopoietic cells (41). Since kinetics, cut-off values and time-points for *WT1* analysis are controversial, its expression should be evaluated in cases without an identifiable molecular and phenotypical marker, and the validated MRD assay developed by ELN should be adopted (42).

## Next-Generation Sequencing (NGS)

Next-Generation Sequencing (NGS) is becoming an important tool for the molecular dissection of AML at the time of initial diagnosis, especially in cytogenetically normal AML, which is characterized by high clonal heterogeneity (43). Indeed, different clones, characterized by specific mutations or their combinations, may show variable sensitivity to therapy and distinct relapse tendency. NGS is potentially applicable to all leukemic patients, but the interpretation of results requires highly specialized bioinformatic approaches. Main NGS technologies include: (a) Whole Genome Sequencing (WGS), (b) Whole-Exome Sequencing (WES), and (c) Targeted-gene sequencing. In particular, the latter method provides simultaneous profiling of several genes of interest and is clinically applicable to dissect the impact of combined mutations as potential targets for MRD assessment, and as measurable biomarkers of treatment (1, 44). Klco et al. performed WES at the time of diagnosis in 71 AML patients, treated with standard induction chemotherapy, and showed that mutations persisting at day 30 in at least 5% of BM cells were associated with reduced overall survival (OS) and increased risk of relapse (45). The NGS-based MRD assessment can also identify potentially important changes occurring at the subclonal level during the disease course (36, 46, 47).

The persistence of molecular MRD at the time of CR as an independent prognostic factor for survival (48), was also demonstrated by Jongen-Lavrencic et al. (49), who analyzed by targeted-NGS 482 AML patients, at diagnosis and in CR after induction therapy. Mutations persisted in about 50% of patients at the time of CR and the presence of most mutations was associated with an increased risk of relapse (50). However, some of the persisting mutations such as *DNMT3A, ASXL1*, and *TET2* (49), collectively termed DTA, known to be frequent in clonal hematopoiesis (CHIP) (51, 52), did not have a prognostic role. It is therefore, clear that NGS-based MRD assessment is hampered by the presence of clonal hematopoiesis and ancestral clones. In this context, our group (53) recently demonstrated that expression levels of *DNMT3A* mutations do not reflect disease status in AML, and that, distinct from *NPM1* mutations, they characterize pre-leukemic populations persisting at the time of CR. Similarly, Debarri et al. (54) showed that MRD-positivity for *DNMT3A* mutations by NGS did not correlate with increased relapse risk during follow-up. Collectively, these data confirm that DTA mutations are not sufficient *per se* to induce leukemia and do not reflect the presence of a specific disease, but may set the genetic ground for the development of myeloid malignancies, mainly in older patients (55).

In addition to the role in MRD assessment, the detection of small subclones by NGS is important to evaluate clonal evolution during the disease course. Actually, some of the mutations are not stable and can become detectable or disappear at the time of relapse, due to the emergence of new resistant subclones, as in the case of the *FLT3*-ITD aberration (47, 56–58). These mutations represent an issue in AML since *FLT3*-ITD-mutated cells, although chemosensitive at diagnosis and responsive to initial chemotherapy, lead to relapse in a substantial number of patients especially those who have not received allo-SCT. Several researchers attempted to develop MRD tools to evaluate *FLT3*-ITD kinetics in AML, but the applicability of this approach was limited because of low sensitivity, and the necessity to design patient-specific assays based on the length of nucleotide inserts (47). On the other hand, MRD negativity for *FLT3*-ITD mutation is not a reliable disease marker due to the occurrence of *FLT3*-ITD negative relapses in about 25% of patients with a positive test at diagnosis (57, 59). Recently, Levis and co-authors (60) described the development of a combined PCR/NGS methodology, with high sensitivity and specificity for *FLT3*-ITD-mutated AML. Although validation in clinical trials is necessary, this tool may represent a good NGS-based MRD platform to evaluate clinical responses, and to trace the clonal evolution of *FLT3-*ITD mutations, taking into account possible phenotypic “shifts” during disease evolution.

Newer molecular alterations are currently evaluated as targets for MRD assessment. Kohlmann et al. successfully used amplicon-based NGS to detect and quantify *RUNX1* gene mutations in a cohort of 814 AML patients. *RUNX1*-mutated transcript levels significantly correlated to clinical outcome, and in particular, stratification of patients according to median *RUNX1* mutation levels influenced event-free survival (EFS) and OS (61). *RUNX1*-MRD longitudinal assessment could be particularly useful to monitor disease progression from myelodysplastic syndromes to secondary AML (9, 61, 62).

Conventionally, although NGS-based MRD measurements could be applicable in all AML patients, its current error rates set the sensitivity level at about 1% (63). Moreover, although several studies underline the potential of NGS to track MRD, technical issues remain to be evaluated. Indeed, many NGS libraries are prepared through multiple rounds of PCR amplification and therefore potential artifacts can be introduced, making it difficult to distinguish these errors from true mutations present at low allele frequency. To overcome this problem, which impairs wide application of NGS, random barcodes or molecular indexes have been introduced in several NGS platforms, to remove errors introduced by PCR amplification, thereby allowing reliable and accurate quantification of genetic targets (63, 64). These molecules, which anneal to specific sequence fragments, may be applied to all NGS-based assays to increase the sensitivity of the detection limits, and to accurately identify low-frequency alleles and copy number variants (65, 66).

## Digital Droplet PCR (ddPCR)

In the clinical practice and research, MRD evaluation requires the ability to identify disease related mutations present at very low frequencies. Digital droplet PCR (ddPCR) is a recently introduced molecular assay with great potential for MRD monitoring, due to its high sensitivity and specificity. It is a high-throughput technology that, unlike conventional RT-qPCR, produces an absolute quantification, by amplifying target genes without a reference standard curve (32, 67). Indeed, although RT-qPCR assays are nowadays carefully standardized for accurate molecular quantifications (31), PCR amplification bias can influence reaction efficiency, leading to imprecise genetic quantification. The ddPCR technique is increasingly applied for the detection of mutations and translocations at high sensitivity, based on patient's genetic characteristics. Starting material for ddPCR may be RNA or DNA molecules that are fractionated into thousands of droplets, where each PCR amplification of the target gene occurs. Compared to NGS, ddPCR is characterized by an inferior error rate, is faster and does not require a bioinformatics expert to analyse the results. Despite its high sensitivity (>10-3), the major pitfall of ddPCR is that a single assay needs to be developed for specific base changes in the same gene.

Among clinically relevant mutations, those affecting *NPM1* are suitable for sensitive ddPCR testing. Actually, *NPM1*-mutated monitoring is sometimes troublesome due to the presence of several frameshift insertions and lack of information on the mutated sequence at diagnosis. In fact, MRD analysis in *NPM1*-mutated patients requires identification of the specific insertion by DNA sequencing to design the appropriate allele-specific RT-qPCR test (38), and requires commercial plasmid standards for each mutation, currently available only for the three most common mutations. Conversely, ddPCR does not require a standard amplification curve and is highly concordant with RT-qPCR for the detection of rare *NPM1* mutations (68). A recent study showed that ddPCR can be used to monitor MRD using multiple *NPM1* mutation-specific primers (69). The multiplex assay has an overall excellent concordance with single mutation-specific ddPCR assays, as well as with conventional RT-qPCR. These data suggest that a single ddPCR can effectively quantify *NPM1* MRD, reducing the potential difficulties associated with *NPM1* quantification, also in patients with unknown or rare mutant sequences.

In addition to *NPM1*, genes involved in the regulation of DNA methylation as *DNMT3A* and *IDH1/2* are recurrently mutated in AML (70), and have been reported to occur very early during leukemogenesis (71). These alterations, in particular in the *DNMT3A* gene, are frequently detectable in the peripheral blood of healthy individuals, representing pre-leukemic clones, in the context of CHIP (51). In AML, Ivey and colleagues (29), analyzed by ddPCR the kinetics of *DNMT3A* and *IDH* mutations in a large cohort of AML patients, who were in molecular remission for the *NPM1*-mutation. They showed that *DNMT3A* and *IDH* alterations may persist in patients in remission at long-term, confirming that these mutations are not disease-specific and may be eliminated by allo-SCT only. Although the prognostic value of *IDH* mutations is still unclear (72), Ferret et al. (73) demonstrated that ddPCR monitoring of *IDH mutations* after allo-SCT, could help to early identify disease persistence and anticipate hematologic relapse. These data have been confirmed by Winters et al. who demonstrated the utility of ddPCR for post-SCT MRD monitoring (74). Finally, although the prognostic value of conventional RT-qPCR in APL is well-established (75), Brunetti et al. recently demonstrated that ddPCR may also be used to monitor patients at high-risk of relapse. In APL, we showed that ddPCR may detect mutations associated with arsenic trioxide (ATO) resistance such as the *PML* A216V mutation (76). We were able to show that this technology could identify mutated cases earlier in the disease course and at very low *PML-RARA* copy number, as compared to conventional sequencing approaches. The identification of the *PML* A216V mutation by ddPCR in APL cases at the time of molecular relapse may in the future help to anticipate treatment decisions in ATO-resistant patients.

## Multiparametric Flow-Cytometry (MFC)

The advantages of MFC for MRD assessment include its applicability to virtually all patients (>90% of AML), the rapidity, and the ability to distinguish viable cells from debris and dead cells (77–80). *Furthermore, the technique is considered less labor-intensive with a shorter turnaround time as compared to RT-qPCR* *(**81**). The availability of instruments equipped with multiple lasers allows the fast implementation of multi-color assays, eventually increasing sensitivity that can be reasonably put between 10*^−3^ *and 10*^−5^.

*The detection of residual leukemic cells by MFC relies on the detection of antigens and/or physical abnormalities that are absent or infrequent in normal BM. Such aberrancies are mainly represented by cross-lineage expression, overexpression, reduced, absent, and asynchronous antigens expression and are defined as leukemia associated immunophenotypes or LAIPs*. A complementary technique of analysis is represented by the “different from normal” (DfN) approach. In this approach, a standardized combination of antibodies is applied to all MRD analyses, regardless of LAIP, allowing the identification of events outside the maturation patterns of normal BM. The harmonization of these analytical strategies has been endorsed by the ELN group (24) and may overcome the concerns on immunophenotypical shifts, that make MRD a moving target in AML (82). The application of panels including at least eight colors and the acquisition of a proper number of events minimizes the possibility to miss minor populations present at diagnosis that may eventually generate relapse (24). The panel of the ELN MRD working party suggests that to achieve a reliable estimation with a threshold set at 0.1%, the amount of residual leukemic cells by MFC should be determined on a denominator of at least 0.5-1 × 10^6^ cells, excluding debris and CD45 negative cells (23, 24).

Nevertheless, the major drawback of MFC-MRD is that it has been defined by few laboratories with a specific and robust expertise (23, 37). This is related in part to differences in specificity of the different LAIPs/DfNs used in clinical practice, which require high levels of expression for the identification of AML-specific events (80).

The value of MRD by MFC has been retrospectively validated as a biomarker of mCR quality with a remarkable impact on DFS, OS, and cumulative incidence of relapse (CIR) (37). Few published studies have addressed this issue prospectively. *In the pediatric protocol AML02, risk-categories were discriminated relying on the upfront genetic/cytogenetic profile and on the level of FCM-MRD after the first cycle of chemotherapy. Treatment was intensified by gemtuzumab ozogamicin in patients with high levels of MRD. The study came out with a superior clinical outcome as compared to previous not risk-adapted trials of the same institution* *(**83*, *84**). In the GIMEMA adult AML1310 trial, patients with intermediate-risk AML (intermediate karyotype or FLT3-TKD positive or c-kit mutated CBF-positive) received autologous-SCT or allo-SCT depending on the level of FCM-MRD, considering a threshold of 0.035%. OS and disease-free survival (DFS) at 24 months were superimposable (78.6 and 61.4% in MRD-positive and 69.8 and 66.6% in MRD-negative patients). These results suggest that whereas in intermediate risk patients, allo-SCT can be avoided if MRD is not detectable, in MRD positive ones allo-SCT can actually prolong OS and DFS up to those of the MRD-negative category* *(**85**)*.

The occurrence of relapse in a proportion of patients achieving a MRD-negative status, roughly ranging between 20 and 25%, still represents a major drawback of all MRD studies (86). This evidence, despite MRD negativity, refines the relapse risk much better than mCR, and still limits its large-scale use in the decision-making process of AML treatment. The possible explanations of this inaccuracy may reside both on technical and biological reasons. In fact, poor quality of BM samples and contamination of peripheral blood and/or immature populations in the regenerative post-chemotherapy phase may hamper the specificity of immunophenotypic combinations adopted to track MRD. *A further explanation may be the presence of minority populations undetectable with current phenotypical approaches (e.g., leukemic stem cells, LSC) that may survive chemotherapy and eventually lead to relapse* *(**84*, *87**). Both normal stem cells and LSC reside in the CD34*+*/CD38– cells and MFC can distinguish LSC by applying a multicolor analysis including a specific set of markers* *(**88*–*91**). When the presence of LSC, at diagnosis or during the treatment course, was determined and compared with MRD the presence of LSC represented a further negative prognosticator both in MRD-positive and, remarkably, in the MRD-negative populations of patients. This observation was confirmed both in a retrospective study, and prospectively in the context of HOVON/SAKK 102 study* *(**89*, *92**). Accordingly, ELN guidelines suggest the inclusion of MFC determination of residual LSC during treatment of AML, suggesting that a dedicated test tube should be developed to this purpose* *(**24*, *84**)*.

*Possible future developments in MRD detection by MFC will be accomplished by the use of high-throughput methods, e.g., next-generation flow cytometry (NGF)* *(**84**). The potential advantage of these techniques is that they can process huge amounts of immunophenotypically stained cell (*>*10*^7^ *cells) in parallel, substantially increasing the level of sensitivity of the conventional 8–10-color MFC assays. Due to their technical features, these techniques may be prospectively repeated along the treatment course, providing an estimate of clonal evolution of the disease. This may prove useful in AML, whose over time intra-clonal variability may hamper the possibility of a real-time estimation of disease plasticity. NGF has been reported to show significant utility in the monitoring of tumor burden in the setting of multiple myeloma* *(**93**)* *and acute lymphoblastic leukemia* *(**94**), suggesting that similar approaches may be exploited in AML*.

Another promising technique in leukemia diagnostics is mass-cytometry time-of-flight (CyTOF). In CyTOF, antibodies are labeled with transition elements instead of fluorochromes, allowing up to 130 different parameters to be combined in the evaluation of a single cell. Furthermore, since rare elements are absent in biological systems, this approach virtually eliminates the background noise, which commonly affects MFC assays (95). Nowadays, although continuous instrument development and the need of complex analytical tools (e.g., SPADE or ViSNE) still limit the wide application of CyTOF to MRD detection on a routine basis, the multiparametricity of this technique may represent the ideal approach to empower our understanding of the heterogeneity of AML also in MRD studies.

## Conclusions

Minimal Residual Disease (MRD) detection has strong clinical relevance in AML and new technological advances have increased its sensitivity and specificity, making it an essential tool in the overall strategy adopted to treat AML. However, different methods have specific indications and require highly specialized expertises (Table 1). Whatever method is indicated in specific situations, especially in the routine clinical care, it is mandatory to use standardized techniques, apply rigorous bioinformatic analyses and define specific time-points, developed in the context of laboratory networks, which can ensure interlaboratory reproducibility. Several techniques may be needed, but the results should be integrated in a final laboratory report, which covers the different methodologies and maximizes clinically useful information, with the final goal of better addressing personalized treatment approaches.

**Table 1:** Applications and potential pitfalls of MRD testing in AML.

**Type of leukemia**	**Marker**	**Method**	**Sensitivity**	**Source**	**Applicability**	**Optimal time point**	**Advantages**	**Pitfalls**
AML with recurrent genetic abnormalities	*PML-RARA*	RT-qPCR	10^−5^ to 10^−6^	B	Specific AML subgroups	- After two cycles of standard induction/consolidation -After the end of treatment -Every 3 months for 24 months after end of treatment	-High stability -Specific -Low background in normal cells	Stable, molecular relapse is a treatment indication
	*NPM1*-mut			PB and/or BM every 4–6 weeks				
	*CBFB-MYH11*							Present in pts in CCR. Transcript kinetics useful
	*RUNX1-RUNX1T1*							
Other AML	*DNMT3A*-mut	ddPCR	10^−3^	BM		To be defined		Unreliable markers, associated with CHIP
	*IDH1/IDH2*-mut						
	*WT1*	RT-qPCR	10^−5^ to 10^−6^	PB		- After standard induction	-Suggested in pts without a MRD-marker.	-Low sensitivity and specificity -Expressed by normal cells
	LAIP	MPFC	10^−4^ to 10^−5^	BM	Wide (>90%)	- After the 2 cycle	-Fast, cost-effective -Single cell analysis	-Subclone expansion -Phenotypic shifts -Complex analysis

## Dedication

Dedicated to our dear mentor and friend Prof. Francesco Lo-Coco.

## Author Contributions

MV, TO, and FB planned and wrote the manuscript. AV, SL, LM, and FL-C reviewed and approved the manuscript.

### Conflict of Interest Statement

The authors declare that the research was conducted in the absence of any commercial or financial relationships that could be construed as a potential conflict of interest.

## References

[1] Papaemmanuil E Gerstung M Bullinger L Gaidzik VI Paschka P Roberts ND. Genomic classification and prognosis in acute myeloid leukemia. N Engl J Med. (2016) 374:2209–21. 10.1056/NEJMoa1516192 27276561 PMC4979995

[2] Voigt P Reinberg D Genomic and epigenomic landscapes of adult *de novo* acute myeloid leukemia the cancer genome atlas research network. N Engl J Med. (2013) 368:2059–74. 10.1056/NEJMoa1301689 23634996 PMC3767041

[3] O'Donnell MR Tallman MS Abboud CN Altman JK Appelbaum FR Arber DA. Acute myeloid leukemia, version 2.2013. J Natl Compr Canc Netw. (2013) 11:1047–55. 10.6004/jnccn.2013.0127 24029121 PMC4161234

[4] Grimwade D Hills RK Moorman AV Walker H Chatters S Goldstone AH. Refinement of cytogenetic classification in acute myeloid leukemia: determination of prognostic significance of rare recurring chromosomal abnormalities among 5876 younger adult patients treated in the United Kingdom Medical Research Council trials. Blood. (2010) 116:354–65. 10.1182/blood-2009-11-254441 20385793

[5] Grimwade D Hills RK. Independent prognostic factors for AML outcome. Hematol Am Soc Hematol Educ Progr. (2009) 385–95. 10.1182/asheducation-2009.1.385 20008224

[6] Döhner H Estey E Grimwade D Amadori S Appelbaum FR Büchner T. Diagnosis and management of AML in adults: 2017 ELN recommendations from an international expert panel. Blood. (2017) 129:424–47. 10.1182/blood-2016-08-733196 27895058 PMC5291965

[7] Döhner H Estey EHE Amadori S Appelbaum FRFR Büchner T Burnett AK. Diagnosis and management of acute myeloid leukemia in adults: recommendations from an international expert panel, on behalf of the European LeukemiaNet. Blood. (2010) 115:453–74. 10.1182/blood-2009-07-235358 19880497

[8] Gale RE Green C Allen C Mead AJ Burnett AK Hills RK. The impact of FLT3 internal tandem duplication mutant level, number, size, and interaction with NPM1 mutations in a large cohort of young adult patients with acute myeloid leukemia. Blood. (2008) 111:2776–84. 10.1182/blood-2007-08-109090 17957027

[9] Daver N Schlenk RF Russell NH Levis MJ. Targeting FLT3 mutations in AML: review of current knowledge and evidence. Leukemia. (2019) 33:299–312. 10.1038/s41375-018-0357-9 30651634 PMC6365380

[10] Cornelissen JJ Gratwohl A Schlenk RF Sierra J Bornhauser M Juliusson G Racil Z Rowe JM Russell N Mohty M. The European LeukemiaNet AML Working Party consensus statement on allogeneic HSCT for patients with AML in remission: an integrated-risk adapted approach. Nat Rev Clin Oncol. (2012) 9:579–90. 10.1038/nrclinonc.2012.150 22949046

[11] Koreth J Schlenk R Kopecky KJ Honda S Sierra J Djulbegovic BJ. Allogeneic stem cell transplantation for acute myeloid leukemia in first complete remission: systematic review and meta-analysis of prospective clinical trials. JAMA. (2009) 301:2349–61. 10.1001/jama.2009.813 19509382 PMC3163846

[12] Burnett AK Goldstone A Hills RK Milligan D Prentice A Yin J Curability of patients with acute myeloid leukemia who did not undergo transplantation in first remission. J Clin Oncol. (2013) 31:1293–301. 10.1200/JCO.2011.40.5977 23439754

[13] Stone RM Mandrekar SJ Sanford BL Laumann K Geyer S Bloomfield CD. Midostaurin plus Chemotherapy for Acute Myeloid Leukemia with a *FLT3* Mutation. N Engl J Med. (2017) 377:454–64. 10.1056/NEJMoa1614359 28644114 PMC5754190

[14] Altman JK Foran JM Pratz KW Trone D Cortes JE Tallman MS. Phase 1 study of quizartinib in combination with induction and consolidation chemotherapy in patients with newly diagnosed acute myeloid leukemia. Am J Hematol. (2018) 93:213–21. 10.1002/ajh.24974 29139135 PMC6586071

[15] Arber DA Orazi A Hasserjian R Borowitz MJ Le Beau MM Bloomfield CD Cazzola M Vardiman JW. The 2016 revision to the World Health Organization classification of myeloid neoplasms and acute leukemia. Blood. (2016) 127:2391–2406. 10.1182/blood-2016-03-643544 27069254

[16] Hussaini MO Mirza A-S Komrokji R Lancet J Padron E Song J. Genetic landscape of acute myeloid leukemia interrogated by next-generation sequencing: a large cancer center experience. Cancer Genomics Proteomics. (2018) 15:121–6. 10.21873/cgp.20070 29496691 PMC5892606

[17] Komanduri KV Levine RL. Diagnosis and therapy of acute myeloid leukemia in the era of molecular risk stratification. Annu Rev Med. (2016) 67:59–72. 10.1146/annurev-med-051914-021329 26473413 PMC5701748

[18] Dohner H Weisdorf DJ Bloomfield CD. Acute myeloid leukemia. N Engl J Med. (2015) 373:1136–52. 10.1056/NEJMra1406184 26376137

[19] Hourigan CS Gale RP Gormley NJ Ossenkoppele GJ Walter RB. Measurable residual disease testing in acute myeloid leukaemia. Leukemia. (2017) 31:1482–90. 10.1038/leu.2017.113 28386105

[20] Grimwade D Freeman SD. Defining minimal residual disease in acute myeloid leukemia: which platforms are ready for “prime time”? Blood. (2014) 124:3345–55. 10.1182/blood-2014-05-577593 25049280

[21] Grimwade D Jovanovic JV Hills RK Nugent EA Patel Y Flora R. Prospective minimal residual disease monitoring to predict relapse of acute promyelocytic leukemia and to direct pre-emptive arsenic trioxide therapy. J Clin Oncol. (2009) 27:3650–8. 10.1200/JCO.2008.20.1533 19506161

[22] Platzbecker U Middeke JM Sockel K Herbst R Wolf D Baldus CD. Measurable residual disease-guided treatment with azacitidine to prevent haematological relapse in patients with myelodysplastic syndrome and acute myeloid leukaemia (RELAZA2): an open-label, multicentre, phase 2 trial. Lancet Oncol. (2018) 19:1668–79. 10.1016/S1470-2045(18)30580-1 30442503

[23] Buccisano F Maurillo L Schuurhuis GJ Del Principe MI Di Veroli A Gurnari C. The emerging role of measurable residual disease detection in AML in morphologic remission. Semin Hematol. (2019) 56:125–30. 10.1053/j.seminhematol.2018.09.001 30926088

[24] Schuurhuis GJ Heuser M Freeman S Béné M-C Buccisano F Cloos J Minimal/measurable residual disease in AML: consensus document from ELN MRD Working Party. Blood. (2018) 131:1275–1291. 10.1182/blood-2017-09-801498 29330221 PMC5865231

[25] Cicconi L Lo-Coco F. Current management of newly diagnosed acute promyelocytic leukemia. Ann Oncol Off J Eur Soc Med Oncol. (2016) 27:1474–81. 10.1093/annonc/mdw171 27084953

[26] Sanz MA Grimwade D Tallman MS Lowenberg B Fenaux P Estey EH. Management of acute promyelocytic leukemia: recommendations from an expert panel on behalf of the European LeukemiaNet. Blood. (2009) 113:1875–91. 10.1182/blood-2008-04-150250 18812465

[27] Jourdan E Boissel N Chevret S Delabesse E Renneville A Cornillet P. Prospective evaluation of gene mutations and minimal residual disease in patients with core binding factor acute myeloid leukemia. Blood. (2013) 121:2213–23. 10.1182/blood-2012-10-462879 23321257

[28] Corbacioglu A Scholl C Schlenk RF Eiwen K Du J Bullinger L. Prognostic impact of minimal residual disease in CBFB-MYH11-positive acute myeloid leukemia. J Clin Oncol. (2010) 28:3724–9. 10.1200/JCO.2010.28.6468 20625124

[29] Ivey A Hills RK Simpson MA Jovanovic JV Gilkes A Grech A. Assessment of Minimal Residual Disease in Standard-Risk AML. N Engl J Med. (2016) 374:422–33. 10.1056/NEJMoa1507471 26789727

[30] Schnittger S Schoch C Kern W Mecucci C Tschulik C Martelli MF. Nucleophosmin gene mutations are predictors of favorable prognosis in acute myelogenous leukemia with a normal karyotype. Blood. (2005) 106:3733–9. 10.1182/blood-2005-06-2248 16076867

[31] Gabert J Beillard E van der Velden VHJ Bi W Grimwade D Pallisgaard N. Standardization and quality control studies of “real-time” quantitative reverse transcriptase polymerase chain reaction of fusion gene transcripts for residual disease detection in leukemia–a Europe Against Cancer program. Leukemia. (2003) 17:2318–57. 10.1038/sj.leu.2403135 14562125

[32] Ravandi F Walter RB Freeman SD. Evaluating measurable residual disease in acute myeloid leukemia. Blood Adv. (2018) 2:1356–66. 10.1182/bloodadvances.2018016378 29895626 PMC5998930

[33] Willekens C Blanchet O Renneville A Cornillet-Lefebvre P Pautas C Guieze R. Prospective long-term minimal residual disease monitoring using RQ-PCR in RUNX1-RUNX1T1-positive acute myeloid leukemia: results of the French CBF-2006 trial. Haematologica. (2016) 101:328–35. 10.3324/haematol.2015.131946 26635039 PMC4815724

[34] Yin JAL O'Brien MA Hills RK Daly SB Wheatley K Burnett AK. Minimal residual disease monitoring by quantitative RT-PCR in core binding factor AML allows risk stratification and predicts relapse: results of the United Kingdom MRC AML-15 trial. Blood. (2012) 120:2826–35. 10.1182/blood-2012-06-435669 22875911

[35] Falini B Mecucci C Tiacci E Alcalay M Rosati R Pasqualucci L. Cytoplasmic nucleophosmin in acute myelogenous leukemia with a normal karyotype. N Engl J Med. (2005) 352:254–66. 10.1056/NEJMoa041974 15659725

[36] Krönke J Bullinger L Teleanu V Tschürtz F Gaidzik VI Kühn MWM. Clonal evolution in relapsed NPM1-mutated acute myeloid leukemia. Blood. (2013) 122:100–108. 10.1182/blood-2013-01-479188 23704090

[37] Ossenkoppele G Schuurhuis GJ. MRD in AML: does it already guide therapy decision-making? Hematol Am Soc Hematol Educ Progr. (2016) 2016:356–65. 10.1182/asheducation-2016.1.356 27913502 PMC6142473

[38] Gorello P Cazzaniga G Alberti F Dell'Oro MG Gottardi E Specchia G. Quantitative assessment of minimal residual disease in acute myeloid leukemia carrying nucleophosmin (NPM1) gene mutations. Leuk Off J Leuk Soc Am Leuk Res Fund. (2006) 20:1103–8. 10.1038/sj.leu.2404149 16541144

[39] Schnittger S Kern W Tschulik C Weiss T Dicker F Falini B. Minimal residual disease levels assessed by NPM1 mutation-specific RQ-PCR provide important prognostic information in AML. Blood. (2009) 114:2220–31. 10.1182/blood-2009-03-213389 19587375

[40] Forghieri F Comoli P Marasca R Potenza L Luppi M. Minimal/measurable residual disease monitoring in npm1-mutated acute myeloid leukemia: a clinical viewpoint and perspectives. Int J Mol Sci. (2018) 19:3492. 10.3390/ijms19113492 30404199 PMC6274702

[41] Hokland P Ommen HB. Towards individualized follow-up in adult acute myeloid leukemia in remission. Blood. (2011) 117:2577–84. 10.1182/blood-2010-09-303685 21097673

[42] Cilloni D Renneville A Hermitte F Hills RK Daly S Jovanovic JV. Real-time quantitative polymerase chain reaction detection of minimal residual disease by standardized WT1 assay to enhance risk stratification in acute myeloid leukemia: a European LeukemiaNet study. J Clin Oncol. (2009) 27:5195–201. 10.1200/JCO.2009.22.4865 19752335

[43] Ley TJ Mardis ER Ding L Fulton B McLellan MD Chen K. DNA sequencing of a cytogenetically normal acute myeloid leukaemia genome. Nature. (2008) 456:66–72. 10.1038/nature07485 18987736 PMC2603574

[44] Papaemmanuil E Gerstung M Bullinger L Gaidzik VI Paschka P Roberts ND Genomic and epigenomic landscapes of adult *de novo* acute myeloid leukemia. N Engl J Med. (2013) 368:2209–21. 10.1056/NEJMoa1301689 PMC3767041 23634996

[45] Klco JM Miller CA Griffith M Petti A Spencer DH Ketkar-Kulkarni S. Association between mutation clearance after induction therapy and outcomes in acute myeloid leukemia. JAMA. (2015) 314:811–22. 10.1001/jama.2015.9643 26305651 PMC4621257

[46] Nazha A Cortes J Faderl S Pierce S Daver N Kadia T. Activating internal tandem duplication mutations of the fms-like tyrosine kinase-3 (FLT3-ITD) at complete response and relapse in patients with acute myeloid leukemia. Haematologica. (2012) 97:1242–5. 10.3324/haematol.2012.062638 22532519 PMC3409823

[47] Ottone T Zaza S Divona M Hasan SK Lavorgna S Laterza S. Identification of emerging FLT3 ITD-positive clones during clinical remission and kinetics of disease relapse in acute myeloid leukaemia with mutated nucleophosmin. Br J Haematol. (2013) 161:533–40. 10.1111/bjh.12288 23480665

[48] Schlenk RF. Is there justification for 4 cycles of consolidation therapy in AML? Best Pract Res Clin Haematol. (2016) 29:341–4. 10.1016/j.beha.2016.10.008 27890257

[49] Jongen-Lavrencic M Grob T Hanekamp D Kavelaars FG Al Hinai A Zeilemaker A. Molecular minimal residual disease in acute myeloid leukemia. N Engl J Med. (2018) 378:1189–99. 10.1056/NEJMoa1716863 29601269

[50] Ravandi F. Is it time to routinely incorporate MRD into practice? Best Pract Res Clin Haematol. (2018) 31:396–400. 10.1016/j.beha.2018.09.013 30466755

[51] Genovese G Kähler AK Handsaker RE Lindberg J Rose SA Bakhoum SF. Clonal hematopoiesis and blood-cancer risk inferred from blood DNA sequence. N Engl J Med. (2014) 371:2477–87. 10.1056/NEJMoa1409405 25426838 PMC4290021

[52] Zink F Stacey SN Norddahl GL Frigge ML Magnusson OT Jonsdottir I. Clonal hematopoiesis, with and without candidate driver mutations, is common in the elderly. Blood. (2017) 130:742–52. 10.1182/blood-2017-02-769869 28483762 PMC5553576

[53] Ottone T Alfonso V Iaccarino L Hasan SK Mancini M Divona M. Longitudinal detection of DNMT3A^R882H^transcripts in patients with acute myeloid leukemia. Am J Hematol. (2018) 93:E120–3. 10.1002/ajh.25061 29417611

[54] Debarri H Lebon D Roumier C Cheok M Marceau-Renaut A Nibourel O IDH1/2 but not DNMT3A mutations are suitable targets for minimal residual disease monitoring in acute myeloid leukemia patients: a study by the acute leukemia french association. Oncotarget. (2015) 6:42345–53. 10.18632/oncotarget.5645 26486081 PMC4747230

[55] McKerrell T Park N Moreno T Grove CS Ponstingl H Stephens J. Leukemia-associated somatic mutations drive distinct patterns of age-related clonal hemopoiesis. Cell Rep. (2015) 10:1239–45. 10.1016/j.celrep.2015.02.005 25732814 PMC4542313

[56] Shih L-Y Huang C-F Wu J-H Lin T-L Dunn P Wang P-N. Internal tandem duplication of FLT3 in relapsed acute myeloid leukemia: a comparative analysis of bone marrow samples from 108 adult patients at diagnosis and relapse. Blood. (2002) 100:2387–92. 10.1182/blood-2002-01-0195 12239146

[57] Kottaridis PD Gale RE Frew ME Harrison G Langabeer SE Belton AA The presence of a FLT3 internal tandem duplication in patients with acute myeloid leukemia (AML) adds important prognostic information to cytogenetic risk group and response to the first cycle of chemotherapy: analysis of 854 patients from the United King. Blood. (2001) 98:1752–9. 10.1182/blood.v98.6.1752 11535508

[58] Angelini DF Ottone T Guerrera G Lavorgna S Cittadini M Buccisano F. A leukemia-associated CD34/CD123/CD25/CD99+ immunophenotype identifies FLT3-mutated clones in acute myeloid leukemia. Clin Cancer Res. (2015) 21:3977–85. 10.1158/1078-0432.CCR-14-3186 25957287

[59] Kottaridis PD Gale RE Linch DC. Prognostic implications of the presence of FLT3 mutations in patients with acute myeloid leukemia. Leuk Lymphoma. (2003) 44:905–13. 10.1080/1042819031000067503 12854887

[60] Levis MJ Perl AE Altman JK Gocke CD Bahceci E Hill J. A next-generation sequencing–based assay for minimal residual disease assessment in AML patients with *FLT3* -ITD mutations. Blood Adv. (2018) 2:825–31 10.1182/bloodadvances.2018015925 29643105 PMC5916006

[61] Kohlmann A Nadarajah N Alpermann T Grossmann V Schindela S Dicker F. Monitoring of residual disease by next-generation deep-sequencing of RUNX1 mutations can identify acute myeloid leukemia patients with resistant disease. Leukemia. (2013) 28:129–37. 10.1038/leu.2013.239 23958918

[62] Dicker F Haferlach C Sundermann J Wendland N Weiss T Kern W Mutation analysis for RUNX1, MLL-PTD, FLT3-ITD, NPM1 and NRAS in 269 patients with MDS or secondary AML. Leukemia. (2010) 24:1528–32. 10.1038/leu.2010.124 20520634

[63] Kohlmann A Grossmann V Nadarajah N Haferlach T. Next-generation sequencing - feasibility and practicality in haematology. Br J Haematol. (2013) 160:736–53. 10.1111/bjh.12194 23294427

[64] Shiroguchi K Jia TZ Sims PA Xie XS. Digital RNA sequencing minimizes sequence-dependent bias and amplification noise with optimized single-molecule barcodes. Proc Natl Acad Sci USA. (2012) 109:1347–52. 10.1073/pnas.1118018109 22232676 PMC3268301

[65] Kivioja T Vaharautio A Karlsson K Bonke M Enge M Linnarsson S. Counting absolute numbers of molecules using unique molecular identifiers. Nat Methods. (2011) 9:72–4. 10.1038/nmeth.1778 22101854

[66] Ahn J Hwang B Young Kim H Jang H Kim H-P Han S-W. Asymmetrical barcode adapter-assisted recovery of duplicate reads and error correction strategy to detect rare mutations in circulating tumor DNA. Sci Rep. (2017) 7:46678. 10.1038/srep46678 28462938 PMC5411960

[67] Coltoff A Houldsworth J Keyzner A Renteria AS Mascarenhas J. Role of minimal residual disease in the management of acute myeloid leukemia-a case-based discussion. Ann Hematol. (2018) 97:1155–67. 10.1007/s00277-018-3330-9 29704019

[68] Bacher U Dicker F Haferlach C Alpermann T Rose D Kern W. Quantification of rare NPM1 mutation subtypes by digital PCR. Br J Haematol. (2014) 167:710–4. 10.1111/bjh.13038 25039748

[69] Mencia-Trinchant N Hu Y Alas MA Ali F Wouters BJ Lee S. Minimal residual disease monitoring of acute myeloid leukemia by massively multiplex digital PCR in patients with NPM1 mutations. J Mol Diagn. (2017) 19:537–48. 10.1016/j.jmoldx.2017.03.005 28525762 PMC5500824

[70] Im AP Sehgal AR Carroll MP Smith BD Tefferi A Johnson DE. DNMT3A and IDH mutations in acute myeloid leukemia and other myeloid malignancies: associations with prognosis and potential treatment strategies. Leukemia. (2014) 28:1774–83. 10.1038/leu.2014.124 24699305 PMC4234093

[71] Welch JS Ley TJ Link DC Miller CA Larson DE Koboldt DC. The origin and evolution of mutations in acute myeloid leukemia. Cell. (2012) 150:264–78. 10.1016/j.cell.2012.06.023 22817890 PMC3407563

[72] Medeiros BC Fathi AT DiNardo CD Pollyea DA Chan SM Swords R. Isocitrate dehydrogenase mutations in myeloid malignancies. Leukemia. (2017) 31:272–81. 10.1038/leu.2016.275 27721426 PMC5292675

[73] Ferret Y Boissel N Helevaut N Madic J Nibourel O Marceau-Renaut A. Clinical relevance of IDH1/2 mutant allele burden during follow-up in acute myeloid leukemia. A study by the French ALFA group. Haematologica. (2018) 103:822–9. 10.3324/haematol.2017.183525 29472349 PMC5927984

[74] Winters A Goosman M Stevens BM Purev E Smith C Pollyea DA Tracking of AML-associated mutations via droplet digital PCR is predictive of outcomes post-transplant. Blood. (2018) 132:2138 10.1182/blood-2018-99-110834

[75] Brunetti C Anelli L Zagaria A Minervini A Minervini CF Casieri P. Droplet digital PCR is a reliable tool for monitoring minimal residual disease in acute promyelocytic leukemia. J Mol Diagn. (2017) 19:437–44. 10.1016/j.jmoldx.2017.01.004 28268092

[76] Alfonso V Iaccarino L Ottone T Cicconi L Lavorgna S Divona M. Early and sensitive detection of PML-A216V mutation by droplet digital PCR in ATO-resistant acute promyelocytic leukemia. Leukemia. (2019) 33:1527–30. 10.1038/s41375-018-0298-3 30651632

[77] Buccisano F Maurillo L Spagnoli A Del Principe MI Fraboni D Panetta P. Cytogenetic and molecular diagnostic characterization combined to postconsolidation minimal residual disease assessment by flow cytometry improves risk stratification in adult acute myeloid leukemia. Blood. (2010) 116:2295–303. 10.1182/blood-2009-12-258178 20548095

[78] Freeman SD Virgo P Couzens S Grimwade D Russell N Hills RK. Prognostic relevance of treatment response measured by flow cytometric residual disease detection in older patients with acute myeloid leukemia. J Clin Oncol. (2013) 31:4123–31. 10.1200/JCO.2013.49.1753 24062403

[79] Terwijn M van Putten WLJ Kelder A van der Velden VHJ Brooimans RA Pabst T. High prognostic impact of flow cytometric minimal residual disease detection in acute myeloid leukemia: data from the HOVON/SAKK AML 42A Study. J Clin Oncol. (2013) 31:3889–97. 10.1200/JCO.2012.45.9628 24062400

[80] Brooimans RA van der Velden VHJ Boeckx N Slomp J Preijers F te Marvelde JG. Immunophenotypic measurable residual disease (MRD) in acute myeloid leukemia: Is multicentric MRD assessment feasible? Leuk Res. (2019) 76:39–47. 10.1016/j.leukres.2018.11.014 30553189

[81] Ilaria M Principe D Buccisano F Maurillo L Sconocchia G Cefalo M Minimal residual disease in acute myeloid leukemia of adults : determination, prognostic impact and clinical applications. Mediterr J Hematol Infect Dis. (2016) 8:1–13. 10.4084/MJHID.2016.052 PMC5111512 27872732

[82] Zeijlemaker W Gratama JW Schuurhuis GJ. Tumor heterogeneity makes AML a “moving target” for detection of residual disease. Cytom Part B–Clin Cytom. (2014) 86:3–14. 10.1002/cyto.b.21134 24151248

[83] Rubnitz JE Inaba H Dahl G Ribeiro RC Bowman WP Taub J. Minimal residual disease-directed therapy for childhood acute myeloid leukaemia: results of the AML02 multicentre trial. Lancet Oncol. (2010) 11:543–52. 10.1016/S1470-2045(10)70090-5 20451454 PMC3171799

[84] Buccisano F Maurillo L Del Principe MI Di Veroli A De Bellis E Biagi A. Minimal residual disease as a biomarker for outcome prediction and therapy optimization in acute myeloid leukemia. Expert Rev Hematol. (2018) 11:307–13. 10.1080/17474086.2018.1447378 29495904

[85] Venditti A Piciocchi A Candoni A Melillo L Calafiore V Cairoli R MRD-directed therapy for young adults with newly diagnosed acute myeloid leukemia: results of the AML1310 trial of the GIMEMA group. In: 22Th EHA Congress. (2017) p. S111. 10.1182/blood.2018886960 31395600

[86] Paietta E. Consensus on MRD in AML? Blood. (2018) 131:1265–6. 10.1182/blood-2018-01-828145 29567752

[87] Al-Mawali A Leukemic stem cells shows the way for novel target of acute myeloid leukemia therapy. J Stem Cell Res Ther. (2013) 3:4 10.4172/2157-7633.1000151

[88] Pollyea DA Jordan CT. Therapeutic targeting of acute myeloid leukemia stem cells. Blood. (2017) 129:1627–36. 10.1182/blood-2016-10-696039.Seminal 28159738

[89] Terwijn M Zeijlemaker W Kelder A Rutten AP Snel AN Scholten WJ. Leukemic stem cell frequency: a strong biomarker for clinical outcome in acute myeloid leukemia. PLoS ONE. (2014) 9:e107587. 10.1371/journal.pone.0107587F 25244440 PMC4171508

[90] Zeijlemaker W Kelder A Oussoren-Brockhoff YJM Scholten WJ Snel AN Veldhuizen D. A simple one-tube assay for immunophenotypical quantification of leukemic stem cells in acute myeloid leukemia. Leukemia. (2016) 30:439–46. 10.1038/leu.2015.252 26437777

[91] Pollyea DA Gutman JA Gore L Smith CA Jordan CT. Targeting acute myeloid leukemia stem cells: a review and principles for the development of clinical trials. Haematologica. (2014) 99:1277–84. 10.3324/haematol.2013.085209 25082785 PMC4116825

[92] Zeijlemaker W Grob T Meijer R Hanekamp D Kelder A Carbaat-Ham JC. CD34+CD38– leukemic stem cell frequency to predict outcome in acute myeloid leukemia. Leukemia. (2018) 33:1102–12. 10.1038/s41375-018-0326-3 30542144

[93] Flores-Montero J Sanoja-Flores L Paiva B Puig N García-Sánchez O Böttcher S. Next generation flow for highly sensitive and standardized detection of minimal residual disease in multiple myeloma. Leukemia. (2017) 31:2094–03. 10.1038/leu.2017.29 28104919 PMC5629369

[94] Theunissen P Mejstrikova E Sedek L Van Der Sluijs-Gelling AJ Gaipa G Bartels M. Standardized flow cytometry for highly sensitive MRD measurements in B-cell acute lymphoblastic leukemia. Blood. (2017) 129:347–57. 10.1182/blood-2016-07-726307 27903527 PMC5291958

[95] Zeng Z Konopleva M Andreeff M. Single-cell mass cytometry of acute myeloid leukemia and leukemia stem/progenitor cells. Methods Mol Biol. 1633:75–86. 10.1007/978-1-4939-7142-8_5 28735481

